# Preparation of High Purity Crystalline Silicon by Electro-Catalytic Reduction of Sodium Hexafluorosilicate with Sodium below 180°C

**DOI:** 10.1371/journal.pone.0105537

**Published:** 2014-08-25

**Authors:** Yuan Chen, Yang Liu, Xin Wang, Kai Li, Pu Chen

**Affiliations:** Department of Chemical Engineering, University of Waterloo, Waterloo, Ontario, Canada; RMIT University, Australia

## Abstract

The growing field of silicon solar cells requires a substantial reduction in the cost of semiconductor grade silicon, which has been mainly produced by the rod-based Siemens method. Because silicon can react with almost all of the elements and form a number of alloys at high temperatures, it is highly desired to obtain high purity crystalline silicon at relatively low temperatures through low cost process. Here we report a fast, complete and inexpensive reduction method for converting sodium hexafluorosilicate into silicon at a relatively low reaction temperature (∼200°C). This temperature could be further decreased to less than 180°C in combination with an electrochemical approach. The residue sodium fluoride is dissolved away by pure water and hydrochloric acid solution in later purifying processes below 15°C. High purity silicon in particle form can be obtained. The relative simplicity of this method might lead to a low cost process in producing high purity silicon.

## Introduction

In 1823, Berzelius obtained iron-free silicon by reducing SiF4 gas, which came from the heat decomposition of K2SiF6, with red-hot potassium metal above 520°C. From then on, the processes of producing silicon with precursor silicon tetrafluoride or trichlorosilane have been extensively studied.

Current industrial manufacturing of high purity silicon adopts the Siemens method using purified trichlorosilane, SiHCl3. With hydrogen gas, SiHCl3, obtained by converting crude silicon with hydrogen chloride, decomposes and deposits silicon onto high-purity silicon rods and enlarges the rods at 1150°C.

The well-known Stanford Research Institute International (SRI) reduction process involves that purified silicon tetrafluoride (SiF4) gas through fractional distillation is reduced to silicon by metal sodium above 500°C. SiF4 is from the heat decomposition of sodium hexafluorosilicate (Na2SiF6) at 647°C:



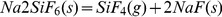
(1)




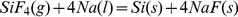
(2)


An alternative method to transform SiF4 gas into elemental silicon with NaAlH4, in which silane (SiH4) gas is decomposed at 727°C to generate elemental silicon, was used by Ethyl Corporation. In 2006, Renewable Energy Corporation announced construction of a plant based on the fluidized bed method using SiH4 gas, which was obtained by conversion of metallurgical grade silicon into SiHCl3 and redistribution/distillation to SiH4. The continuous flow process recycles all hydrogen and chloride materials back to the initial reactors, while continuous distillation steps purify the SiH4 gas.

However, there are many drawbacks with these methods, including high deposition temperature, high cost for constructing durable reactors, high energy consumption, operation with explosive raw materials, and post-treatment of hazardous exhausted gas and amorphous silicon dust waste. Much of the recent research effort to produce solar cell grade silicon has thus focused on electrochemical reduction of silica in molten salts [1]–[4] at 850°C, or metallo-thermic reduction [5]–[7] of silicon compounds. Among them, the magnesio-thermic reduction method [5] above 650°C was well-known.

The preparation of crystalline silicon using other silicon precursors has also been reported. Among them, the synthesis of nanometer-sized silicon crystals by reducing SiCl4 with metal sodium in a nonpolar organic solvent at high temperature (385°C) and high pressure (> 100 atmospheres) was reported [7].

To our knowledge, no studies have been conducted on electrochemical sodium reduction to obtain crystalline silicon using one step process at temperature less than 180°C and in nitrogen atmosphere with a pressure of less than 1 atm. Moreover, this method does not involve silicon precursor gas purification that is necessary to all above-stated industrial processes. The silicon preparation carried out at low temperature may effectively reduce amounts of impurities from side reactions and containers.

## Materials and Methods

Conversion method of sodium hexafluorosilicate into silicon particles by metal sodium was investigated as follow:

Under nitrogen atmosphere, the certain amount of Sodium (a purity of >99wt%, Aldrich) and sodium hexafluorosilicate (Analytical reagent, Alfa Aesar) which had been dried at 120°C for 2 hours to remove the moisture, were put into the round bottom flask with three-necks. The Na_2_SiF_6_: Na molar ratio were <0.25, 0.25, 0.3. One neck of round bottom flask was used as nitrogen gas passage which was through a condenser. One glass pipe was used as gas inlet which was put in the condenser. One magnetic stirrer was put into the round bottom flask. A piece of single-crystal silicon plate (CZ, Phosphorous dopant, Resistivity 1–10 ohm/cm, orient <100>+0.9, Virginia semiconductor) was used as negative working electrode. Another piece of silicon plate was used as positive counter electrode. The two electrodes were put into the round bottom glass flask through the other two necks. Such flask was set in oil bath that was on a fisher hotplate. All experiments were conducted under a dry nitrogen atmosphere. Copper wire and stainless steel clip were used to connect with Si wafers. The silicon wafers were used to contact the Na-Na_2_SiF_6_ mixture. A typical experimental example was as follows: the amount of Sodium was 1.0 g, the amount of Na_2_SiF_6_ was 2.1 g. The mixture of sodium and sodium hexafluorosilicate were stirred at a high speed 750 rpm and at 120°C, 150°C, 180°C, 200°C, or 250°C respectively. A direct current potential 1.4 V, or 2.4 V, or 3.6 V supplied by DC power (B&K precision) was applied to the two electrodes. When Na-Na_2_SiF_6_ mixture was heated at the fixing temperature and stirred at a speed of 750 rpm, they turned dark. After there were brown particle materials appearing on the surface of the glass flask, kept holding at that temperature for half an hour, then stopped the reaction and cooled the mixture to room temperature.

The brown region materials were immersed in ultra-pure water to selectively dissolve sodium hexafluorosilicate and sodium fluoride. Then the samples were exposed to a 1 M hydrochloric acid (HCl) solution to get rid of impurities contained in the samples. The ultra-pure water treatment and the subsequent HCl solution treatment were conducted at 10°C, 40°C, or 60°C respectively. The treatment container was polypropylene beaker. When filtering, whatman filter paper and buchner funnel were used. All experimental operations were done without cleanroom.

Characterization: Scanning electron microscopy was conducted with a field emission scanning electron microscope operating at an accelerating voltage of 20 kV. XRD studies were carried out at 20°C in air atmosphere, using a D8 Discover X-ray diffraction system made by Bruker Company. The purity of the silicon powder was determined by Elan 9000 ICP-MS system (Perkin Elmer Sciex).

## Results and Discussion

The situation that nobody had carried out the preparation of silicon under these conditions was probably due to two preconceived notions: (1) Na_2_SiF_6_ begins to decompose at 647°C [6] and has no melting point; (2) silicon tetrafluoride, which is a byproduct of producing superphosphate fertilizer from phosphate rock [6], is not pure. This was substantiated by the consequences that (1) the temperature for converting Na_2_SiF_6_ into silicon tetrafluoride must be above 647°C; (2) SiF_4_ gas has to be purified by passing it over iron at 797°C to remove air and SO_2_, and by subsequent fractional distillation [6].

However, we found that metal sodium can react with solid Na_2_SiF_6_ below 200°C:



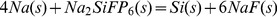
(3)


**Figure 1:**
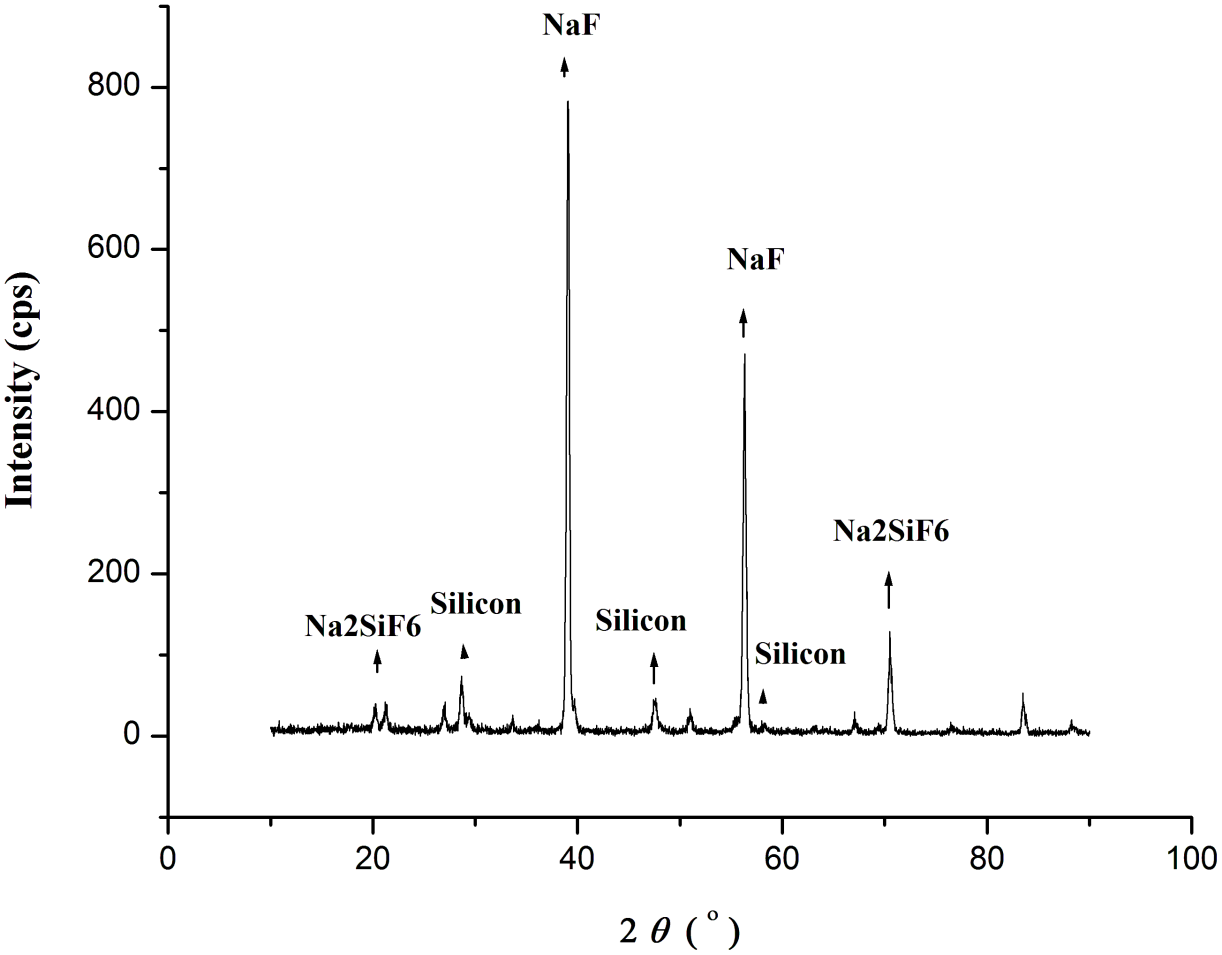
XRD pattern of the produced samples in glass flask.

The experimental set-up and reaction phenomena were described in the Experimental section (also refer to Figure A in File S1). The reaction happened obviously when brown materials in the reactor became visible by eyes. The reacted samples (see Figure B in File S1) were brown colour. The following X-ray diffraction (XRD) analyses indicated that the brown region contained silicon. The unreacted samples were observed to contain three regions of different colours. The region located nearest to the bottom of the glass flask was silver in colour, which was metal sodium. The other two regions, where both metal sodium and Na_2_SiF_6_ existed, were black (containing more metal sodium) and gray (containing more Na_2_SiF_6_), respectively. The XRD analysis of the reacted samples in Figure 1 showed that not only the diffraction peaks of crystalline silicon (JCPDS 27-1402) existed in the XRD pattern, but also those of NaF (JCPDS 36-1455) and a few unreacted Na_2_SiF_6_ (JCPDS 33-1280) (see Figure C in File S1). No peaks of metal sodium or silicon nitride Si_3_N_4_ existed in the XRD pattern.

**Figure 2:**
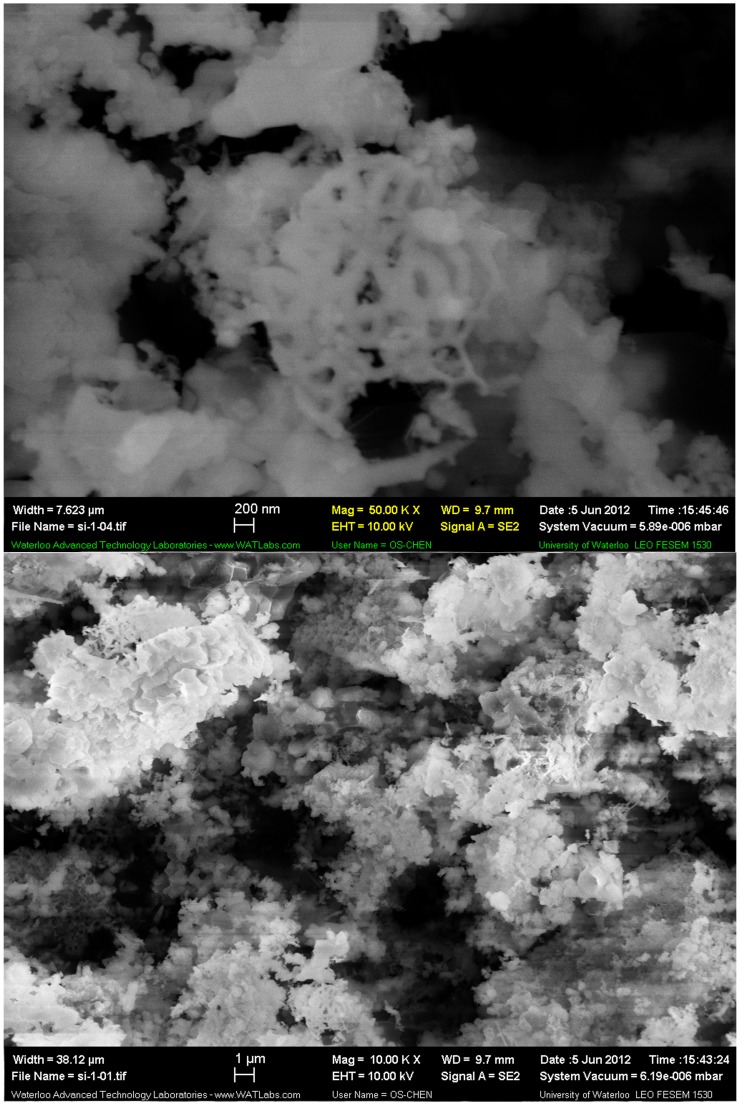
Silicon powder SEM micrographs recorded at different magnification after the sample was washed with pure water.

After the sample (see Figure B in File S1) was washed with pure water, SEM results in Figure 2 showed that the size of silicon particles ranged from several tens of nanometres to 30 micrometres.

Although the free energy of reaction is a quantitative measure, and accurate data for such reactions are not readily available, it is usual for the chemists to rely on the heats of substance formation to estimate the heats of the actual reactions which take place above 25.15°C. The reaction (3) could be thought of as the combination of the reactions (1) and (2), considering similar states of metal sodium. The reaction (2) is strongly exothermic and the reaction (1) is endothermic. Based on the data of the standard Gibbs free energy for substance formation [8], the changes in Gibbs free energy, standard entropy and standard enthalpy for the reaction (3) are −523.6 kJ/mol, −86.9 J/mol•K and −550 kJ/mol, respectively (see Table S1 in File S1). Consequently from the chemical thermodynamic point of view, the reaction (3) could occur spontaneously at the temperature of 30.7°C theoretically, and release heat. This confirms the occurrence of the reaction.

**Figure 3:**
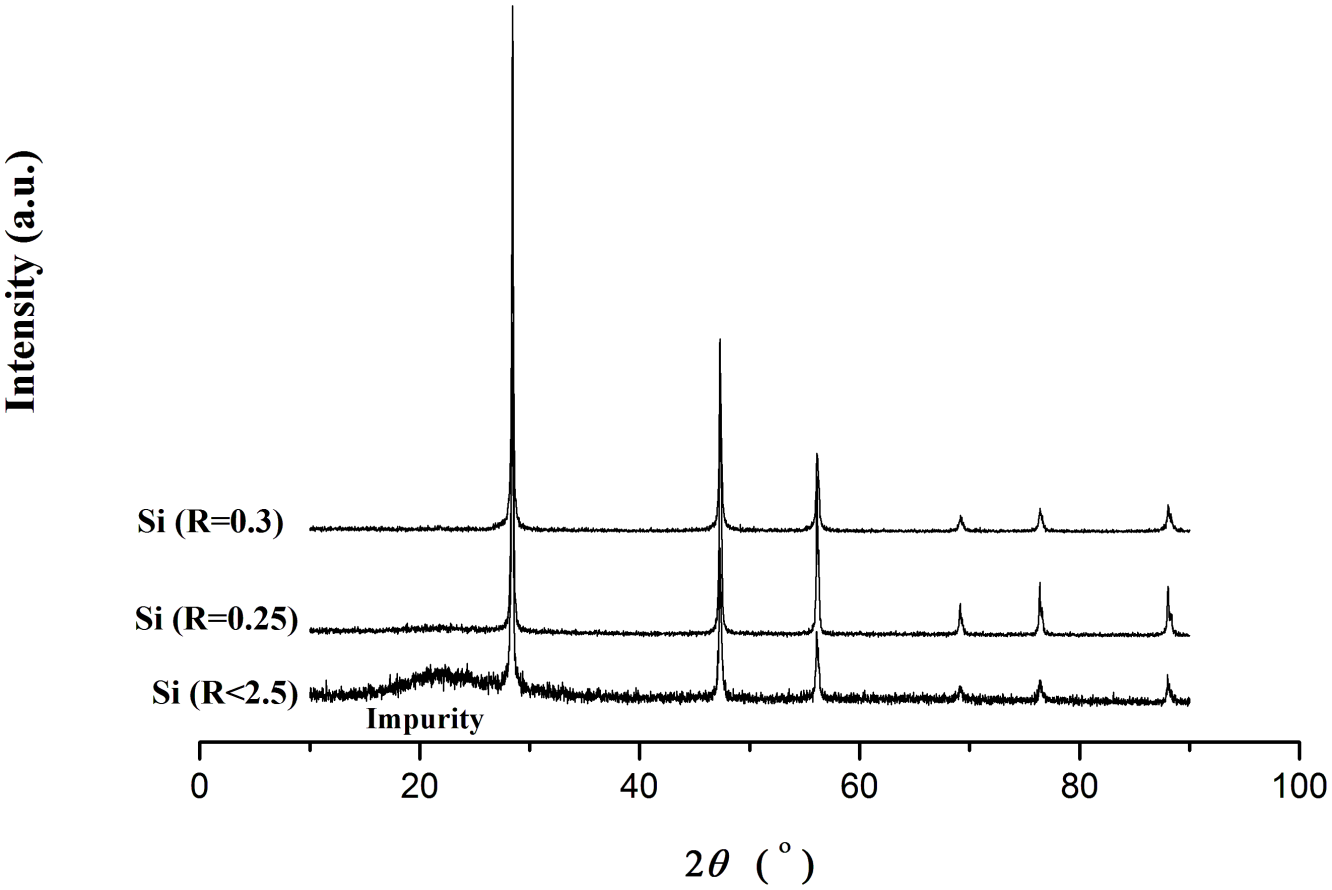
XRD patterns of silicon particles prepared at different ratios of raw materials in glass flask (R is the value of Na2SiF6: Na molar ratio) after the samples were washed with pure water.

If sodium hexafluorosilicate and sodium were stirred at a speed of 750 rpm and maintained at 180°C with applying a 3.6 V electrical voltage to the electrodes, the molar ratio (between 0.25 and 0.35) of Na_2_SiF_6_ to sodium played an important role in the purity of silicon produced. When the sample was immersed in water, there was no reaction of sodium metal with water. This proved that the reaction conversion in terms of sodium was nearly 100%. In Figure 3, the XRD analysis of the produced samples showed that when the Na_2_SiF_6_: Na molar ratio was 0.3∶1, there were fewer impurities in silicon particle samples after washing with pure water. Because sodium metal can react with water to produce NaOH very violently, the residual of sodium metal would affect the purity of silicon and lead to Na_2_SiO_3_ (see Table S1 in File S1).

**Figure 4:**
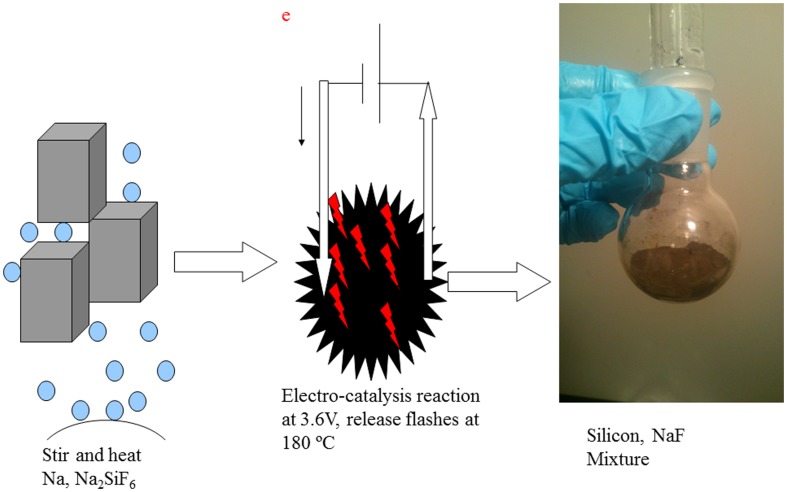
Description of the reaction mechanism.

To evaluate the electrical potential effect control experiments were done without applying potential to the electrodes. The reaction could take place at 200°C with a stirring magnet at a speed of 750 rpm. Dispersing the reactants evenly was important here, and the reaction would not occur without stirring even at 300°C. When the Na_2_SiF_6_: Na molar ratio was suitable (between 0.25 and 0.35), only the brown mixture could be observed (also refer to Figure 4).

When sodium metal was melted, liquid sodium formed a dark conductive dispersion with Na_2_SiF_6_. While applying 2.4 V electrical voltage to the electrodes, the reaction could take place at 190°C and the colour of the liquid-solid mixture changed more quickly than the one without applying potential. Flashes were often observed at the same time as the dark liquid-solid mixture became brown all-solid mixture (also refer to Figure 4). When the potential applied was 3.6 V, the reaction could proceed at below 180°C. Based on the definition of chemical catalysis and electro-catalysis, it is the chemistry in electrochemical reactions, which are most hampered, needing catalytic acceleration. Electrochemical catalysis does not differ very much fundamentally and mechanistically from chemical catalysis, apart from the fact that charge transfer rates and electrosorption equilibriums depend exponentially on electrode potential.

A possible electro-catalytic reduction reaction mechanism is described in Figure 4. The whole reactive systems might involve the exchange of charges between the electrodes and electron donors or acceptors present in the liquid-solid mixture, with ionic charge transfer through the liquid-solid mixture between two electrodes. The flashes might be the result of discharge of short circuits between sodium metal and Na_2_SiF_6_.

The actual decomposition potential should be higher than the ideal reversible decomposition potential (1.36 V) that is estimated by the Nernst equation 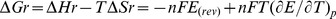 (see Table S1 in File S1) when T is supposed to be 298 K. However, the potential actually required is the sum of the theoretical reversible decomposition voltage, overvoltage at the electrolyte-electrode interphase boundary, and the ionic conduction resistance of the electrolyte. The ionic conduction resistance of the electrolyte depends on the ion diffusion speed (stirring magnet speed affects it greatly), the distance of the electrodes from each other, and the current density. When electrical energy was input to overcome the activation energy threshold of each sub-reaction in the reaction (3), the overall reaction could be accelerated. Once flash occurred, the entire reaction was completed within minutes. However, due to the complication of chemical kinetics in this reaction system, it is difficult to predict the accurate reaction temperature and reaction rate. Note that an increase in temperature might lower the reversible decomposition potential. Na2SiF6 begins to decompose at 920 K (647°C) and has no melting point. This further complicates the reaction kinetics. The experimental results are reported when certain applied potential, stirring speed, concentration of reactants and heating temperature are chosen, and the reaction takes place. Na2SiF6 can decompose below 453 K (180°C) with the presence of sodium metal.

**Figure 5:**
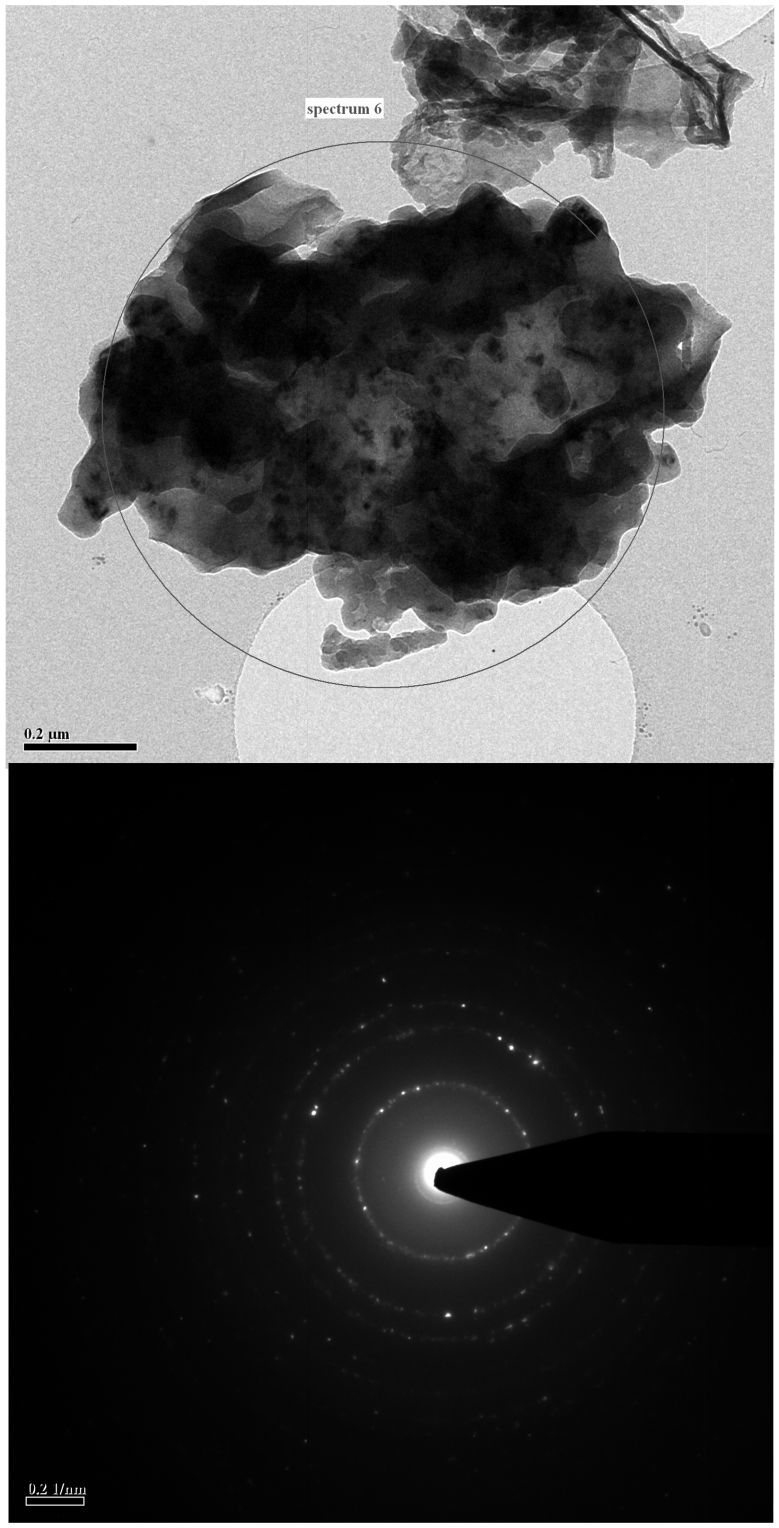
HTEM image and SAED patterns of silicon particles washed with pure water and HCl at 10°C.



To separate silicon from NaF, the samples were dissolved into water to form two immiscible phases, for which solid–liquid extraction (migration of impurities from silicon to NaF solution) provided additional purification. NaF and Na_2_SiF_6_ could be dissolved into ultra-pure water easily. This process is different from the known the SRI process [6], which comprises heating Si-NaF mixture at a temperature slightly below the melting point of silicon with a molten purifying agent, which does not appreciably react with silicon to cause the impurities to separate from silicon. Because the water washing process takes place at room temperature, the possibilities to cause impurities into silicon are reduced greatly. The polycrystalline structure of silicon was confirmed by HTEM and SAED analysis shown in Figure 5. No Na_2_SiF_6_ or NaF (see Figure C in File S1) was found. When trying to obtain ultra-pure silicon through pure water and HCl solution washing under air atmosphere, by comparing the XRD patterns, we observed that surface oxidation of silicon particles could be inhibited below 10°C:

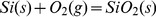
(4)


The change in Gibbs free energy, standard entropy and standard enthalpy for the reaction (4) are −856.3 kJ/mol, −182.5 J/mol•K and −910.7 kJ/mol, respectively [8]. Consequently from the chemical thermodynamic point of view, the reaction (4) can occur spontaneously at 25°C (see Table S1 in File S1).

**Figure 6:**
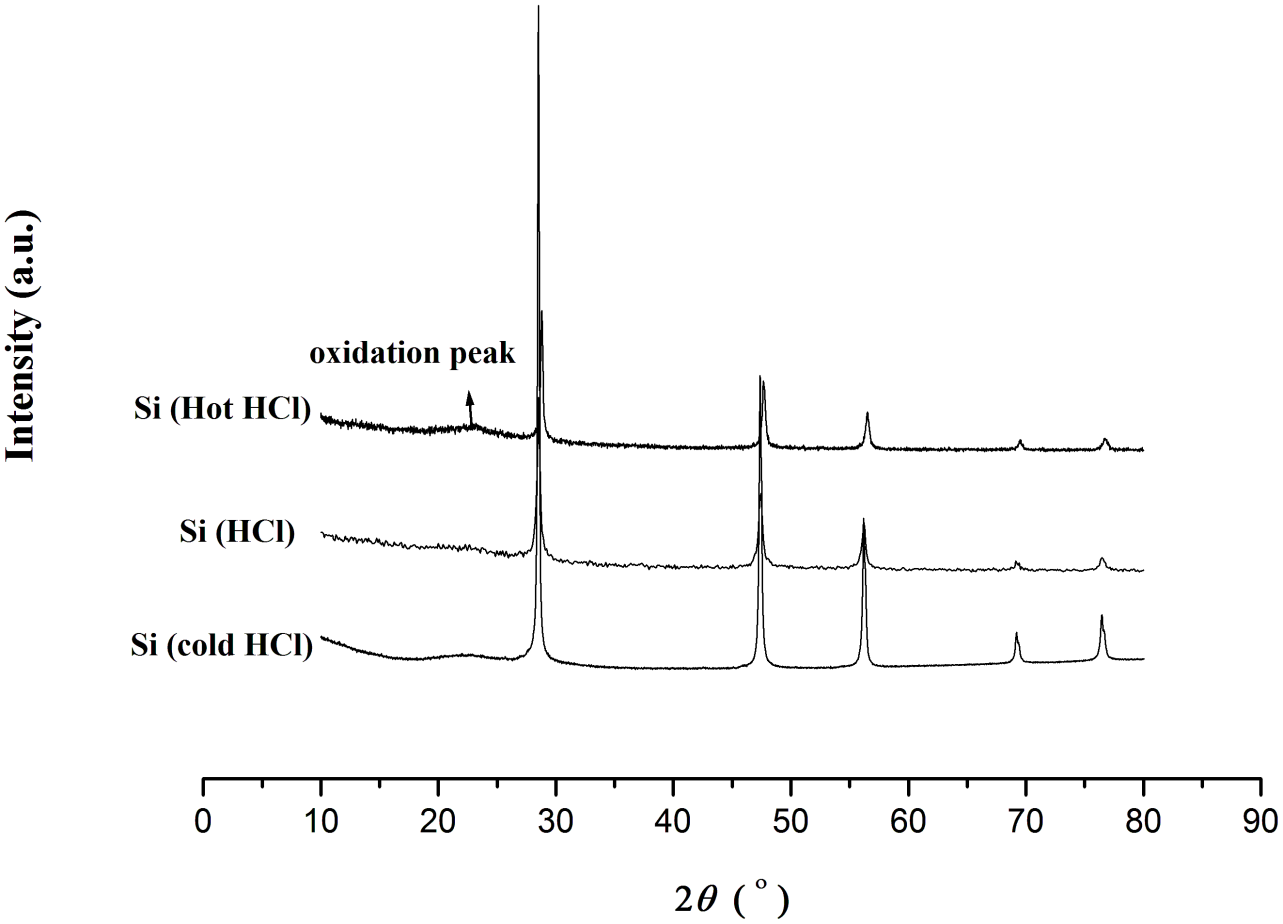
XRD patterns of silicon particles washed with pure water and HCl at 10°C, 40°C and 60°C.

Silicon particles were washed by ultra-pure water and HCl solution at 40°C and 60°C, respectively, in air atmosphere. The results showed that silicon particles could be mildly oxidized at 60°C (see Figure D in File S1) when exposed to aqueous HCl without removing oxygen. Figure 6 showed the XRD pattern of the silicon particles, which were washed at 10°C through ultrapure water and HCl solution. In this XRD pattern, there was no obvious peak near 2θ = 23°, which was corresponding to a broadened peak for amorphous SiO_2_.

The possible reasons are as follows: 1) surface oxidation of silicon might be inhibited at below 10°C; 2) the remainder NaF in the sample can react with aqueous HCl solution to produce HF solution, and the HF solution can remove some silicon dioxide on the surface of silicon particles. According to the magnesiothermic reduction method [5] at 650°C, the silicon dioxide on the surface of silicon particles can be removed by using the ethanol-based hydrofluoric solution. Our results are consistent with the statement that oxygen in water can oxidize silicon slowly at room temperature in the magnesiothermic reduction method [5].

Table 1 showed that the total content of metallic impurities from the silicon powder obtained by ICP-mass was less than 34.86 ppm at. Sodium in the sample was 26 ppm at. Sodium was the major metal impurity. Aluminum and iron contents were 1.6 ppm at and 0.8 ppm at, respectively. Phosphorus in the silicon sample was 0.9 ppm at. This means that the production of solar-grade silicon with phosphorus content of <1 ppm (parts per million) is possible in this method, if the raw material Na_2_SiF_6_ is of analytical grade reagent. The detailed elemental analysis could be seen in the Table S2 in File S1. Since all experimental operations were done without cleanroom, with respect to metallic impurities, the purity of the silicon was estimated to be at least 99.996 at%. In the Siemens method, hydrogen chloride gas etching is generally applied to remove impurities from the surface of silicon rods and can make purity of silicon above 99.99999%. According to Henry's law, the concentration of a gas in a liquid is directly proportional to the partial pressure of the gas in equilibrium with the liquid [6]. Aqueous HCl solution can remove metallic impurities equally well as hydrogen chloride gas in the Siemens method. Therefore, we believe the concentration of metal impurities could be further reduced with increasing the washing times of HCl solution in a clean environment, which avoids contamination of dust from atmosphere.

**Table 1:** Results of Impurities analysis in silicon powder by ICP-MS.

Metallic Impurities in ppm atom	
Na	26
Mg	0.7
Al	1.6
K	0.4
Sc	0.2
Ti	0.5
V	0.1
Mn	1.9
Fe	0.8
Ni	0.5
Cu	0.1
As	0.2
Sr	0.1
Sn	0.2
Sb	0.2
Total	33.2
13impurities:Cr,Zn,Ga,In,Te,La,Pr,Nd,Dy,Hg,Pb Total	<1.3
36impurities:Li,Be,Ca,Ge,Rb,Y,Nb,Ru,Rh, Pd,Cd,Ag,Cs,Ce,Sm,Eu,Gd,Tb,Ho,Er, Tm,Yb,Lu,Hf,Ta,W,Re,Os,Ir,Pt,Au,Tl,Bi,U,Th Total	<0.36
Silicon Purity at %	>99.996
*All other non-metal impurities in ppm atom. Not including H,C,N,B,O,F	
I	1.1
P	0.8
Br	<0.1
S	0.5
Total Conc.	<2.5

Not including H,C,N,B,O,F, Inert gas The detection limit of the analysis was 1 ppm wt., and accuracy and precision were estimated to be on the order of 10% relative.

Compared with current industrial manufacturing of high purity silicon, our new and relatively lower temperature preparation method has several advantages as follows:

First, it does not involve purifying of liquid or gas silicon compounds, and will not consume energy as high as that of the Siemens method, which requires preparation and distillation of trichlorosilane, or the fluidized bed method which requires pure silane. Such purifying steps (involving conveyance, heat transfer, separation and/or mixing operations) in the Siemens method or the fluidized bed method also result in huge equipment cost.

Second, our method produces more silicon in per cubic meter of reactor space at 180°C with applying 3.6 V to electrodes, and saves large amounts of electrical energy because electro-catalytic reduction of Na_2_SiF_6_ could be carried out completely within several minutes in liquid-solid reactive state, not in gas-solid reactive state as used in the Siemens method at 1127°C, or the fluidized bed method at above 647°C. The Siemens method produces poly-silicon at about $28/kg. The reactants in our method are low-cost and cheaper than silicon tetrachloride and hydrogen gas used in the Siemens method. The price of metal sodium (99.7%) is $2.3/kg and that of Na_2_SiF_6_ (99.5%) is $0.6/kg in the world market. There is actually an energy cost saving, comparing with the cost of existing industrial methods that take place above 1100°C. Sodium metal is used as raw material, but this disadvantage could be offset greatly by recycling NaF to prepare Na_2_SiF_6_. Na_2_SiF_6_ can be obtained by adding NaF to H_2_SiF_6_. The product solid silicon can be purified with pure water and HCl solution at room temperature. The cost for preparing high pure silicon is lower.

Third, no gas products or polluting effluents are produced. At the end of the treatment, water only contains Na_2_SiF_6_, which can be easily collected and recycled. It is an eco-friendly process, unlike that of the Siemens method, which requires recycling toxic acid gas, silicon tetrachloride and hydrogen gas.

Its drawback is that it is a batch process rather than a continuous one, so that more washing times by water and HCl solution are required to obtain high pure silicon powder.

## Conclusions

In conclusion, for the first time we demonstrated that sodium hexafluorosilicate powder can be electro-catalytically reduced to pure crystalline silicon at below 180°C by sodium metal. High pure crystalline silicon powder can be obtained by washing with HCl solution at low temperature. Further work to obtain solar grade silicon is undergoing with better preparation conditions. Our study provides a new promising opportunity for low-cost production of high purity crystalline silicon.

## Supporting Information

File S1 **This file contains Figures A, B, C, and D, and Tables S1 and S2.** Figure A. The glass reactor for preparing silicon below 175°C. Figure B. The image of the products of reaction Figure C. XRD pattern of NaF and Na2SiF6. Figure D. EDX analyses obtained from silicon particles washed at 313K.(Carbon is from conductive tape). Table S1. Standard Thermodynamic Properties of Chemical Substances. Table S2. The ICP-Mass test results of silicon samples.
(PDF)
