# Correction: Preparation of High Purity Crystalline Silicon by Electro-Catalytic Reduction of Sodium Hexafluorosilicate with Sodium below 180°C

**DOI:** 10.1371/journal.pone.0117875

**Published:** 2015-02-06

**Authors:** The PLOS ONE Staff

There is an error in the third paragraph of the Introduction and the first equation. The 2 in Na_2_SiF_6_ should be subscripted. The correct sentence and the correct first equation are: The well-known Stanford Research Institute International (SRI) reduction process involves that purified silicon tetrafluoride (SiF_4_) gas through fractional distillation is reduced to silicon by metal sodium above 500°C. SiF_4_ is from the heat decomposition of sodium hexafluorosilicate (Na_2_SiF_6_) at 647°C:

$$Na_{2}SiF_{6}(s)=SiF_{4}(g)+2NaF(s)$$ (1)

In the Results and Discussion, there is an error in the third equation. The letter “P” was inadvertently added to the second value. Please view the complete, correct equation here:

$$4Na(s)+Na_{2}SiF_{6}(s)=Si(s)+6NaF(s)$$ (3)

These errors were introduced during preparation of this manuscript for publication. The publisher apologizes for these errors.
