# Supplementary material for: Preparation of High Purity Crystalline Silicon by Electro-Catalytic Reduction of Sodium Hexafluorosilicate with Sodium below 180°C
Source: PLoS One. 2014 Aug 25;9(8):e105537. doi: 10.1371/journal.pone.0105537 (PMC4143305; doi:10.1371/journal.pone.0105537)
Supplement: File S1 — This file contains Figures A, B, C, and D, and Tables S1 and S2. Figure A. The glass reactor for preparing silicon below 175°C. Figure B. The image of the products of reaction Figure C. XRD pattern of NaF and Na2SiF6. Figure D. EDX analyses obtained from silicon particles washed at 313K.(Carbon is from conductive tape). Table S1. Standard Thermodynamic Properties of Chemical Substances. Table S2. The ICP-Mass test results of silicon samples. (PDF) [file pone.0105537.s001.pdf]

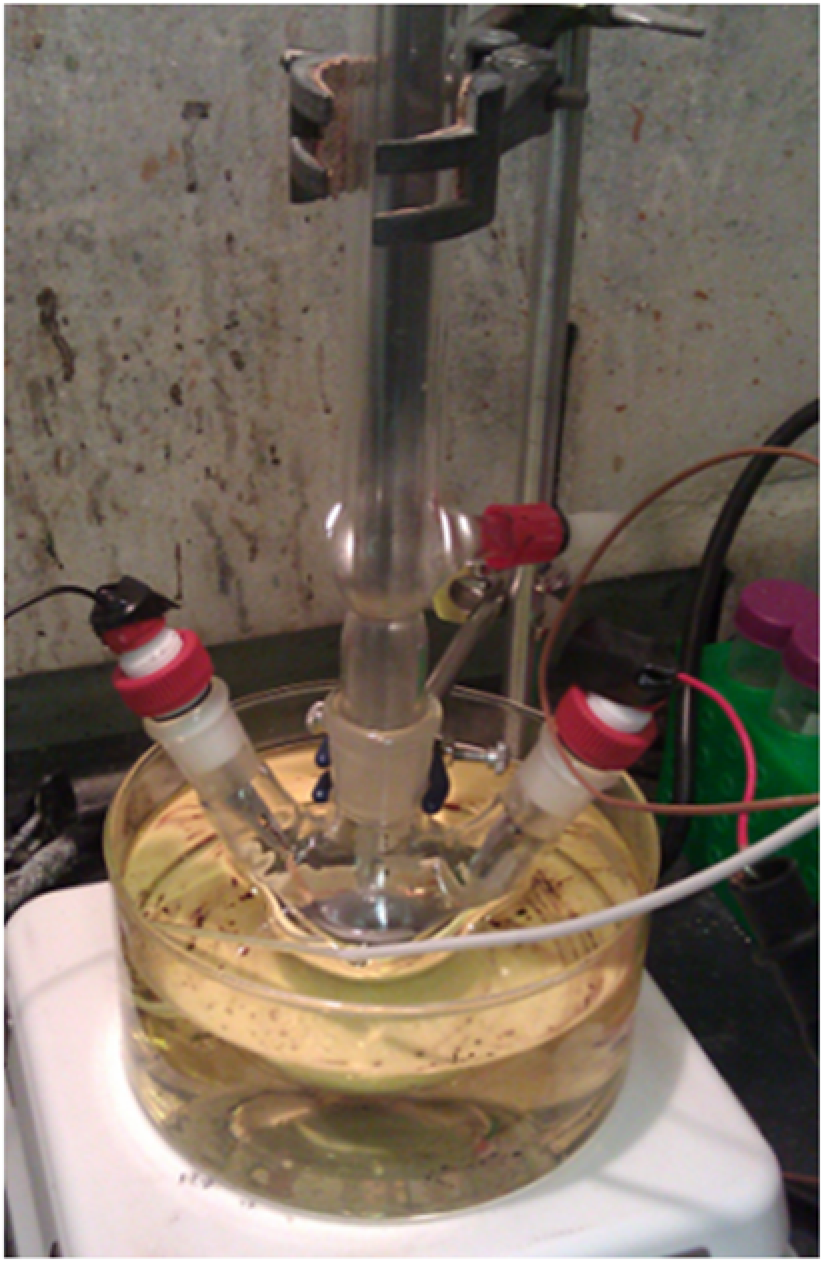

Figure A in File S1. The glass reactor for preparing silicon below 175°C

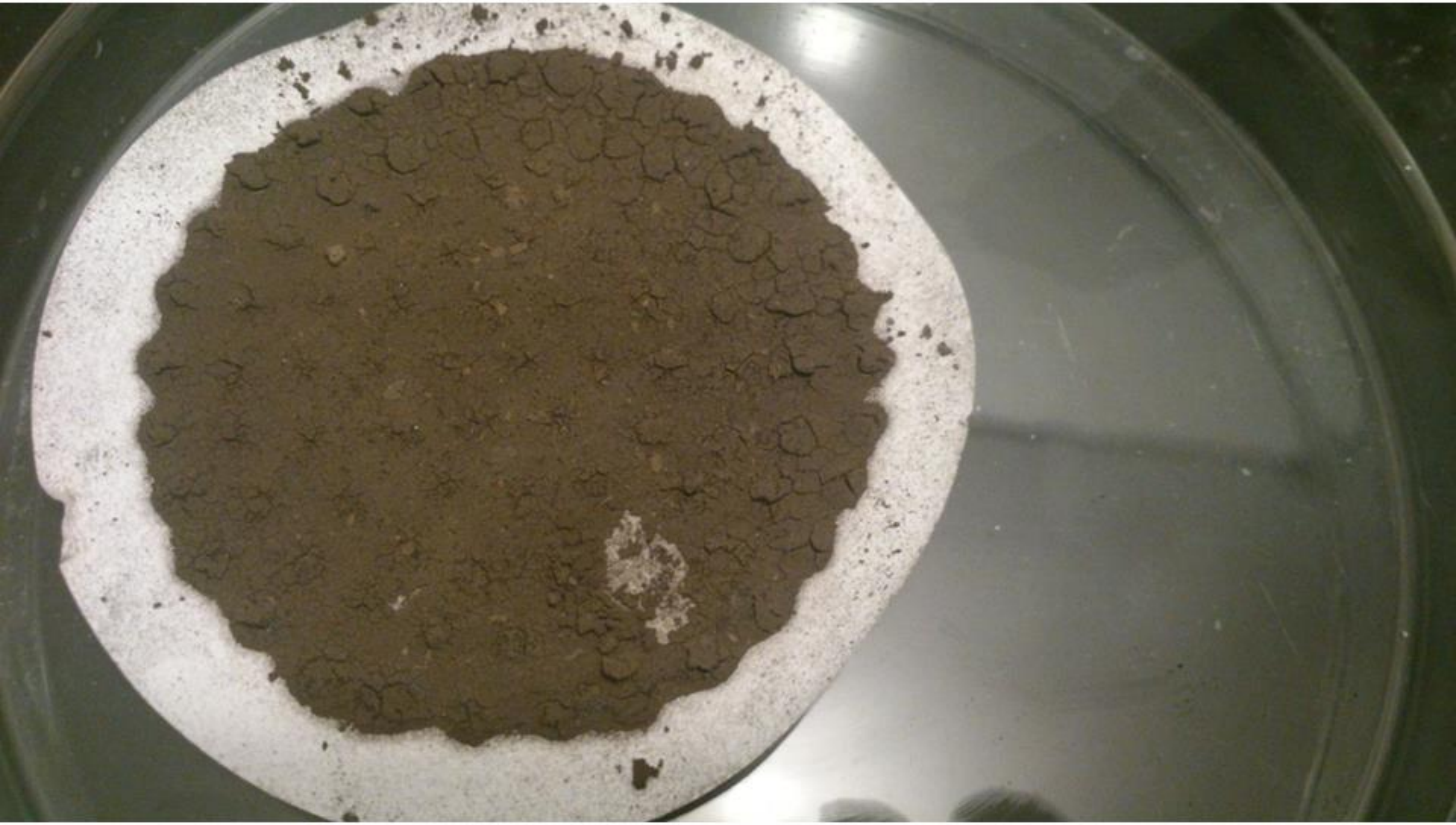

Figure B in File S1. The image of the products of reaction

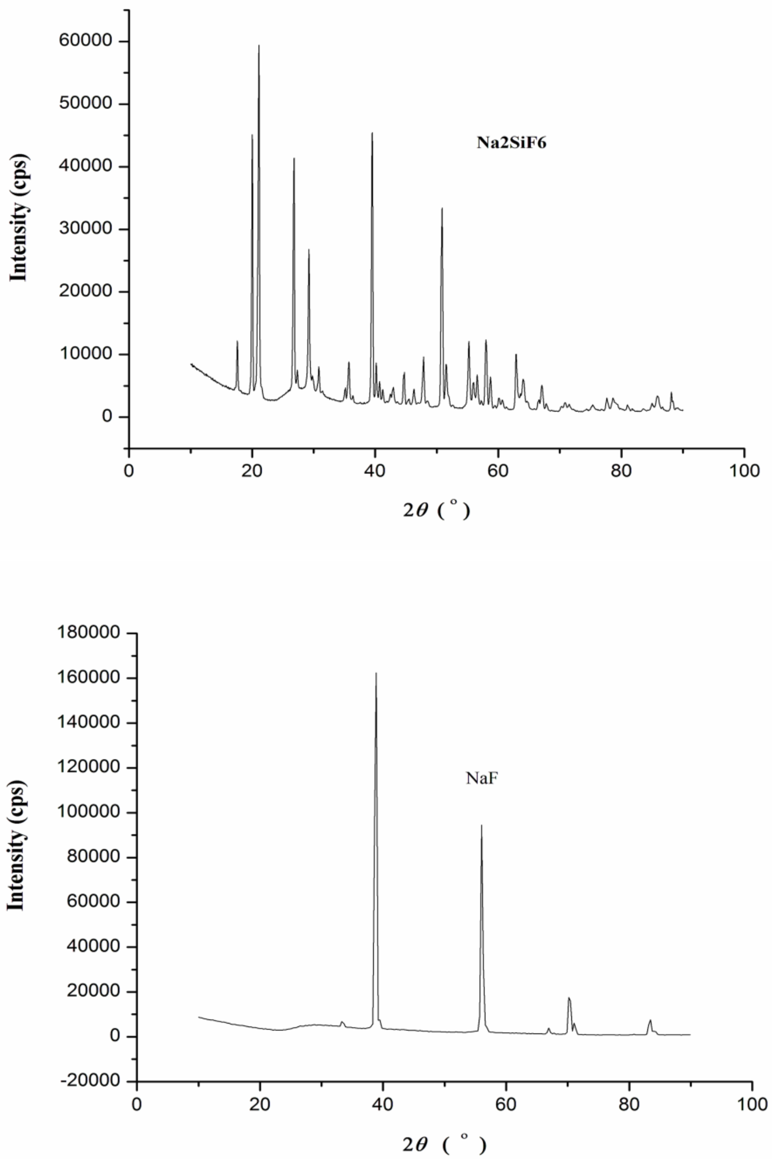

Figure C in File S1.XRD pattern of NaF and Na2SiF6

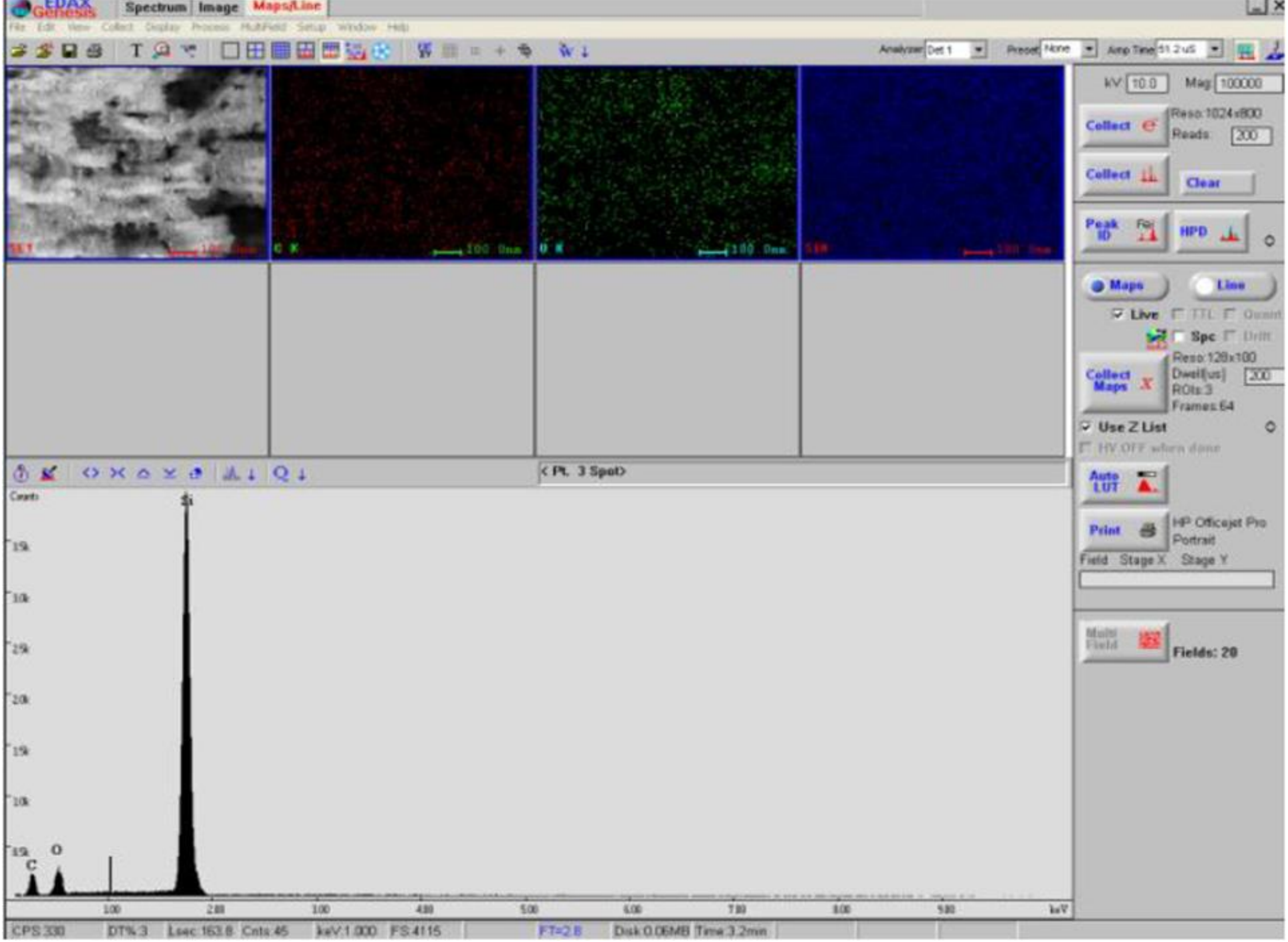

Figure D in File S1.EDX analyses obtained from silicon particles washed at 313K. (Carbon is from conductive tape)

Table S1. Standard Thermodynamic Properties of Chemical Substances

STANDARD THERMODYNAMIC PROPERTIES OF CHEMICAL SUBSTANCES (Gas)				
Molecular formula	$\Delta_f H°$ kJ/mol	Δ_f G°kJ/mol	S° J/mol•K	Cp J/mol•K
Na	107.5	77.0	153.7	20.8
Si	450.0	405.5	168.0	22.3
Si F_4	-1615.0	-1572.8	282.8	73.6
O_2	0.0		205.2	29.4
H_2	0.0		130.7	28.8
STANDARD THERMODYNAMIC PROPERTIES OF CHEMICAL SUBSTANCES (Crystal)				
Molecular formula	$\Delta_f H°$ kJ/mol	Δ_f G° kJ/mol	S° J/mol•K	Cp J/mol•K
NaF	-576.6	-546.3	51.1	46.9
Na_2SiF_6	-2909.6	-2754.2	207.1	187.1
Na	0.0		51.3	28.2
Si	0.0		18.8	20.0
H_2O	-285.8	-237.1	70.0	75.3
NaOH	-425.8	-379.7	64.4	59.5
Na_2SiO_3	-1554.9	-1462.8	113.9	
SiO_2	-910.7	-856.3	41.5	44.4

Reference in *CRC Handbook of Chemistry and Physics*, 92nd Edition, (Ed: W. M. Haynes), CRC Press/Taylor and Francis, Boca Raton, FL. **2012**, Section 5.

$$\Delta G_r = \Delta H_r - T\Delta S_r = -nFE_{(rev)} + nFT(\partial E / \partial T)_p$$

$$Na_2SiF_6(s) = SiF_4(g) + 2NaF(s) \quad (1)$$

ΔG_r= -1572.8+2× (-546.3) - (-2754.2) = 88.8 kJ/mol

$$SiF_4(g) + 4Na(l) = Si(s) + 4NaF(s) \quad (2)$$

$$4Na(s) + Na_2SiF_6(s) = Si(s) + 6NaF(s) \quad (3)$$

ΔG_r=6×(-546.3) + 0- (-2754.2)-0= -523.6kJ/mol
T=303.7K

$$W = \Delta G_r = -nFE_{(rev)}$$

$E_{(rev)}$ =-ΔG_r/nF=523600/(4 ×96495) =1.36V

$$2Na(s) + 2H_2O(\text{l}) = 2NaOH(s) + H_2(g) \quad (4)$$

ΔG_r=2× (-379.7)+0-2× (-237.1)-0=-285.2 kJ/mol

$$H_2O(l) + 2NaOH(s) + Si(s) = 2H_2(g) + Na_2SiO_3(s) \quad (5)$$

ΔG_r=-1462.8+0-2× (-379.7)-0-(-237.1)= -466.3 kJ/mol

$$Si(s) + O_2(g) = SiO_2(\text{s}) \quad (6)$$

ΔG_r=-856.3 kJ/mol
T=298.1K

Table S2. The ICP-Mass test results of silicon samples

Element	Mass	Conc.(parts per thousand)
Li	7	1.60E-06
Be	9	2.00E-06
Na	23	0.021
Mg	24	5.70E-04
Al	27	0.0015
P	31	9.20E-04
S	34	5.90E-04
Cl	37	<2.800E-5
K	39	5.30E-04
Ca	43	1.20E-05
Sc	45	3.60E-04
Ti	47	8.50E-04
V	51	2.20E-04
Cr	53	6.10E-05
Mn	55	0.0037
Fe	57	0.0016
Co	59	1.50E-04
Ni	60	9.30E-05
Cu	63	2.50E-04
Zn	66	1.00E-04
Ga	69	5.50E-05
Ge	72	1.80E-05
As	75	4.60E-04
Se	82	<9.900E-6
Br	79	<4.300E-5
Rb	85	2.30E-05
Sr	88	3.30E-04
Y	89	1.20E-05
Zr	90	3.80E-05
Nb	93	<3.600E-6
Mo	95	1.40E-05
Ru	101	<3.400E-6
Rh	103	<3.700E-6
Pd	105	7.80E-06
Ag	107	1.80E-05

Cd	111	<2.500E-5
In	115	2.00E-04
Sn	118	0.001
Sb	121	9.40E-04
Te	125	2.10E-04
I	127	0.0048
Cs	133	3.30E-05
Ba	137	9.20E-05
La	139	9.00E-05
Ce	140	1.70E-05
Pr	141	2.40E-04
Nd	146	6.90E-05
Sm	147	<4.800E-6
Eu	153	<4.900E-6
Gd	157	<3.600E-6
Tb	159	<3.200E-6
Dy	163	5.60E-06
Ho	165	<2.500E-6
Er	166	9.00E-06
Tm	169	7.90E-06
Yb	172	<1.700E-6
Lu	175	<1.700E-6
Hf	178	<1.500E-6
Ta	181	<1.600E-6
W	182	2.20E-05
Re	185	<1.500E-6
Os	189	<1.200E-6
Ir	193	<1.600E-6
Pt	195	<2.200E-6
Au	197	2.50E-05
Hg	202	3.30E-04
Tl	205	<1.700E-6
Pb	208	1.90E-04
Bi	209	7.10E-05
Th	232	<2.700E-6
U	238	7.90E-06

Testing method: Silicon powder is added in HF (GR) solution to be digested for 2hours in microwave digesting oven. Then H_2SO_4 (MOS grade) is added to the above digested solution. The solution is heated on hotplate to get rid of fluoride. At last use ultrapure water to dilute the remains to be ready for ICP-mass test. The purity of the silicon powder was obtained by one minus the total content of impurities (H,C,N,B,O,F are not included). The purity of the silicon powder was determined by Elan 9000 Perkin Elmer Sciex.

The detection limit of the analysis was 1ppm wt., and accuracy and precision were estimated to be on the order of 10% relative.
